# Determinants of intrauterine contraceptive device utilization at primary health care facilities in Mekelle City, northern Ethiopia

**DOI:** 10.1186/s40834-021-00164-7

**Published:** 2021-07-01

**Authors:** Gebremaryam Temesgen Birgoda, Haftom Gebrehiwot, Sultan Hussen Hebo, Birhane Hagos, Genet Assefa, Negussie Boti Sidamo, Mulugeta Shegaze Shembri

**Affiliations:** 1 Department of Midwifery, College of Medicine & Health Sciences, Arba Minch University, Arba Minch, Ethiopia; 2 Department of Midwifery, College of Health Sciences, Mekele University, Mekele, Ethiopia; 3 Department of Public Health, College of Medicine & Health Sciences, Arba Minch University, Arba Minch, Ethiopia; 4 Department of Midwifery, College of Medicine & Health Sciences, Dilla Minch University, Arba Minch, Ethiopia; 5 Department of Midwifery, College of Medicine & Health Sciences, Wolkite University, Wolkite, Ethiopia

**Keywords:** Intra uterine contraceptive device, Case control, Determinant factors

## Abstract

**Background:**

Each year, the current level of modern contraceptive use averts 188 million unintended pregnancies, which in turn results in 112 million fewer abortions. Of the 867 million women in the developing world who are sexually active and want to avoid becoming pregnant, approximately 222 million of them have an unmet need for modern contraception. In spite of several advantages and potential effectiveness of Intra Uterine Contraceptive Device, its utilization still too low in Sub Saharan African countries including Ethiopia.

**Objectives:**

To identify the determinant factors for utilization of intra uterine contraceptive device among women visiting primary health care facilities in Mekelle city.

**Method:**

Facility based unmatched case-control study design was conducted among 234 women (78 cases and 156 controls). Data was collected by structured questionnaire. Data entry and cleaning was done using EPI- Info version 5.3.1 and analysis done using SPSS version 20.0 statistical software. During analysis the variables were defined, categorized and the difference in variables was determined. Odds ratio used to show degree of association between independent variables with Intra Uterine Contraceptive Device.

**Result:**

Marital status ([AOR (95%CI) =8.59(2.60–28.43)], number of pregnancies (AOR (95%) CI = 5.69(1.020–31.802), number of alive children [AOR (95%CI) =3.5 (1.03–11.9) are variables continued to have statistically significant association with use of Intra Uterine Contraceptive Device. Other determinants found to have significant association includes awareness about Intra Uterine Contraceptive Device, visual exposure to Intra Uterine Contraceptive Device, and participants told about availability of health care provider able to insert Intra Uterine Contraceptive Device.

**Conclusion:**

This study has identified marital status, Gravidity, number of alive children and awareness to Intra Uterine Contraceptive Device as major determinants for use of Intra Uterine Contraceptive Device. Thus, it is vital at addressing the aforementioned determinants will be vital to improve utilization of Intra Uterine Contraceptive Device.

**Plain English summary:**

Among long acting reversible modern contraceptive methods, Intra Uterine Contraceptive Devices (IUCDs) are the most reliable and effective as well as with fewer side effects. Despite these advantages and cost effective potential of Intra Uterine Contraceptive Device its utilization is still too low in Sub Saharan countries like Ethiopia. Thus, this study intended to identify the factors that limit the utilization of Intra Uterine Contraceptive Device among women of Ethiopia in Mekele City. The study identify that the utilization of Intra Uterine Contraceptive Device was determined by the marital status of the women, the number of previous pregnancy and recent alive children and the level of awareness about Intra Uterine Contraceptive Device of the women. Therefore, providers training that focus on promoting Intra Uterine Contraceptive Device, centering on increasing awareness and practice about Intra Uterine Contraceptive Device is very important.

## Introduction

Family planning is defined as the use of contraceptive methods enables couples to have desired number of children, to control the timing and spacing of pregnancy [1]. Modern contraceptives methods are categorized into three: Long acting reversible contraceptive methods which consists of development and introduction of modern intrauterine devices (IUCDs) and Implants, permanent contraceptive methods which includes tubal ligation and vasectomy and short term contraceptives methods constitutes predominant method in developing countries (inject-able), oral pill, male and female condoms, foam tablet and cervical cap [2].

The development and introduction of modern intrauterine devices (IUDs) in 1960, shortly after the advent of oral contraceptives, makes the beginning of the modern era of long-acting reversible contraception. Then rapidly became the method of choice in pioneering programs in Taiwan, Korea, and other countries like United States [3].

It is estimated about 500 million women in the developing world are using some form of family planning. From 113 million married women of reproductive age in sub-Saharan Africa, 21 million (one in five) married women use family planning. 14.7 million less than one of seven) women currently use modern contraception only 2.7 million women use long-acting or permanent contraception [4].

Copper IUCD are the most reliable and effective form of LARC have several advantages over other forms including implanone (it serves us both long and short acting as fertility return immediately after removal, fewer side effect than hormonal methods, fewer discontinuation rate (13%) than other forms oral contraceptives (43.5%),injectable (40.6%) and condom 50.4% over 12 months. It can be inserted immediately after delivery and abortion, has no adverse effect with other medications like anti-retroviral therapy and can be inserted by trained health providers at all level. In spite of these advantages and cost effective potential of IUCD its utilization is still too low only 8% in Sub Saharan countries and 9% in South Central Asia, even as high as 22% in South east Asia [5].

Fertility rate is 2.5 children per woman World fertility pattern (WFP 2015). According to WHO estimate about global MMR is 216 and 800 maternal death every day occur with preventable causes related with pregnancy and child birth. Ninety Nine Percent of this death is in developing countries, Sub-Saharan Africa contributes 66% followed by south Asia [5]. Each year, the current level of modern contraceptive use averts 188 million unintended pregnancies, which in turn results in 112 million fewer abortions, 1.1 million few newborn deaths and 150,000 fewer maternal deaths [6].

Africa remains the region with the highest fertility at 4.7 children per woman, which is the fastest growth from any other region and projected to account 21% of the global population by 2050 [7]. The Federal Democratic Republic of Ethiopia is the second most populous country in sub Saharan Africa with an estimated population of approximately 92.08 million people1 and total fertility rate (TFR) of 4.6 children per woman (2.3 in urban areas and 5.2 in rural areas) and the tenth largest by area with its 1.1 million square kilometers [8].

Globally, 163 million (15% of reproductive aged women use IUCD [9]. Countries with the higher number of IUCD user includes Cuba (43.5%), Vietnam (37.7%), Egypt (36.5%) and China (44.9%) (World Bank Africa Region,), the lowest use of the IUCD is observed in Africa, where few countries show more than one or 2 % use of the method [10].

By now the progress of CPR in Ethiopia is increased from 6% in 2000 to 35% by 2016. However, The CPR is highly dependent on short-term family planning methods (e.g. Nearly 23% for inject able followed by implant 8% and IUCD constitute 2%),and unmet need for family planning is still high for spacing births (13%) and limiting (9%) [11]. The CPR in Tigray is similar with the national Average (35%) in EDHS 2016,and dependent on injectable (19%) followed by implant (11%) and pills (4%),but the prevalence of IUCD (1%),which is below the national level [12].

Recent evidence related to intrauterine contraception provides various assumptions as to the reasons for low and declining use of the IUCD. Among various reasons, incorrect perceptions and knowledge of IUCDs, skills of providers and facility readiness for IUCD service have been considered as a major limitation [13].

Although the IUCD is available free of charge in the public sector services, it is not being utilized adequately. Various studies suggest that lack of clients‟ knowledge and/or misunderstanding about IUCD method, reluctance of service providers in providing information about IUCD, in adequately trained health care providers, and absence of continuing education and awareness of clients have been suggested to be major limiting factors to improve acceptability of this safe and effective contraceptive method of IUCD [14].

Recognizing this situation, the Federal Ministry of Health (FMOH), under Health service development program IV, has set a target CPR of 66% by 2015, besides, the FMOH has considered the important role of long-acting nonpermanent and permanent methods and aims to provide 20% of all family planning clients with these long-acting methods [12]. But it remained un attainable as CPR in the country is 35% which relies on injectable [13].

In general, it is possible to conclude from the above discussions that modern contraceptive use in Ethiopia is dependent on short acting contraceptive methods and IUCD methods share of use remains below or else underutilized in most women in Ethiopia (19, 20). Thus, understanding the factors that limits the use of IUCD in women in Mekelle city, therefore, can help to inform interventions that can strengthen women’s empowerment in ways to plan their pregnancies to achieve desired family size.

## Methods and materials

### Study setting, design and population

Study was done in Mekelle town, which is the capital of Tigray region from October 25–November 25, 2017 where the overall prevalence IUCD in the city is 1.5% (27). Facility based unmatched case-control study design was used. Participant number is determined by using formula for comparison of two population proportions with unequal proportion and EPINFO Version 3.5.3 statistical software. Based on the assumption of level of significance, α = 0.05, Power 1-^β^ = 90%, Control to case ratio, = 2:1 and the study done in Debremarkos town partner discussion about modern contraceptive is determinant factor for utilization of IUCD, in that study women who have discussion are 2.5 times more likely to use CI (0.143,0.833)(62.5% of users and 40% of non-users have partner discussion about contraceptives [2]. Using this reference the calculated sample size become 234(78 IUCD users and 156 non users). It is taken since it increases my sample size.

### Sampling procedures

In the town there are 9 health centers, 2 facilities that are providing reproductive health services (marry stops international and family guidance association) and 2 primary hospitals that facilities are selected randomly and cases are allocated based on their last 2 months provision (August and September, 2017). In each facility, all women who are IUCD users during the study were selected as cases for this study, if the selected case was not fulfill the inclusion criteria, she would be dropped before controls selected. For each case, two controls that used OCPs or injectable during the study were selected after the new IUCD user and if the selected control did not fulfill the inclusion criteria, the next woman who uses OCPs or Injectable during the study were included in the study as a control.

### Data collection procedures and tools

A structured questionnaire containing four parts (Socio-demographic and socio-economic variables, Reproductive variables, Knowledge, practice and, decision making variables and Health system variables) was used to interview study participants. It was prepared in English and translated to Tigrigna language. Questionnaires were pre-test and edited. One day training was given for data collectors. Pretest was done in the next day after training of data collectors at Mekele general hospital which is not the actual study settings. Seven trained female clinical nurses and Midwives collected the data immediately after the client obtained the service (exit interview). Before starting the interview, the data collectors took informed verbal consent from the client to participate in the study and registered the chart number of the client for checking any missed or incomplete data.

During the supervision, quality and completeness of gathered information by the data collectors was checked periodically by the principal investigator and timely corrections made which could help a lot in improving the quality, consistency and completeness of data of subsequent interviews. The collected data cleaned manually by the investigator.

### Data analysis

After manual cleaning, data entry were done using EPI- Info version 3.5.3. and analysis was done using SPSS version 20.0 statistical software. During analysis the variables defined, categorized and recoded then frequencies of the different variables and cross- tabulations determined. Odds ratio used to show statistical significant level of association between independent variables with IUCD utilization at *p*-value < 0.25 and < 0.05 for Binary and Multivariate Logistic Regression respectively.

### Ethical considerations

Ethical clearance was obtained from the Health Research Ethics Review Committee at the college of health sciences. Formal recommendation letter was written from department of Midwifery for Tigray Regional Health bureau (TRHB) in turn TRHB wrote similar letter to each selected PPHC and permission obtained from the main office of selected health facility. The issue of consent, privacy and confidentiality were given due emphasis during the training of data collectors. Informed written consent was sought from all study participants at all levels.

## Result

### Socio-demographic characteristics

A total of 234 women of reproductive age attending the selected Primary health care facilities were interviewed giving response rate of 100%. The age of respondents showing that it is normally distributed at mean (+SD) is 32 ± 6.22 (range 20–43) and 26.28 ± 5.83 (range 17–40) for case and control respectively. Less than 10% of cases and controls can read and write but more than half 52.6% of respondents in each group had secondary education. The following table summarizes the socio-demographic characteristics of the respondents (Table 1).

**Table 1:** Socio-demographic characteristics of clients visiting family planning clinic at Primary Health Care Facilities in Mekelle city. Northern Ethiopia, Tigray region, 2017

Variables	Cases (78)	Controls (156)	Total (234)
Age (years)			
15–24	9 (11.5)	69 (44.3)	78 (33)
25–29	23 (29.5)	42 (26.8)	65 (27)
30–34	17 (21.8)	26 (16.7)	43 (18)
35–39	16 (20.5)	13 (8.3)	29 (12)
40–44	13 (16.7)	6 (3.8)	19 (8)
Religion			
Christian	69 (88.5)	134 (85.9)	171 (73.1)
Muslim	9 (11.5)	22 (14.1)	31 (13.5)
Ethnicity			
Tigray	64 (82.2)	130 (83.3)	194 (82.9)
Amhara	10 (10.6)	19 (12.2)	29 (12.4)
Others	6 (7.7)	12 (7.7)	18 (7.7)
Educational status			
Primary school	10 (12.8)	37 (23.7)	47 (19.3)
Secondary school	41 (52.6)	82 (52.6)	123 (52.6)
Above secondary	27 (34.6)	37 (23.7)	64 (27.4)
Marital status			
Married	73 (93.6)	101 (64.7)	174 (74.4)
Single	5 (6.4)	55 (35.3)	60 (25.6)

### Reproductive health characteristics of the women

Ninety Seven Point Four percent of cases and Seventy Five Point Six Percent of controls experienced pregnancy. More than half 56.6% of cases experienced pregnancy 3 to 4 times and less than 10% of controls were pregnant for 5 and more times. Among those who had history of pregnancy, the majority of cases 72(98.6%) and controls 105(93.8%) had history of normal birth. Majority 58(80.6%) of cases and 82(78.1%) of controls were planned to space birth. All of the case have no history of STI, but 15(9.6%) of controls had STIs history having treated for all of them (Table 2).

**Table 2:** Reproductive health characteristics of clients visiting family planning clinic at Primary Health Care Facilities in Mekelle city. Northern Ethiopia, Tigray region, 2017

Variables	Cases(78)	Controls(156)	Total (234)
History of pregnancy (234)			
Have experienced pregnancy	76 (97.4)	118 (75.6)	194 (82.9)
No Experience of Pregnancy	2 (2.6)	38 (24.4)	40 (17.1)
Number of pregnancy (*N* = 194)			
1 to 2 times	19 (25)	67 (57.35)	86 (44.6)
3 to 4 times	43 (56.6)	40 (34.2)	83 (43)
≥5 times	14 (18.4)	10 (8.5)	24 (12.4)
History of abortion (*N* = 194)			
Experienced abortion	37 (48.7)	56 (47.9)	93 (48.2)
No experience of abortion	39 (51.3)	61 (52.1)	100 (51.8)
Number of Abortion (*N* = 93)			
Experienced Abortion Once	32 (86.5)	51 (91.2)	83 (89.4)
Experienced Abortion Twice	5 (13.5)	5 (8.8)	10 (10.6)
History of birth (*N* = 194)			
experienced birth	72 (98.6)	105 (93.8)	177 (95.7)
No experience of birth	1 (1.45)	7 (6.2)	8 (4.3)
Number of Birth (*N* = 177)			
Once	6 (8.2)	33 (31.4)	38 (21.9)
Twice	22 (30.1)	30 (28.6)	52 (29.2)
>Twice	45 (61.6)	42 (40)	87 (48.9)
Number of alive children (*N* = 177)			
1 child	6 (8.2)	33 (31.4)	39 (21.9)
2 children	25 (34.2)	30 (28.6)	55 (30.9)
> 2 children	42 (57.5)	42 (40)	4 (47.2)
Future plan of fertility (234)			
Spacing Birth	58 (80.6)	82 (78.1)	140 (79.1)
Limiting Birth	14 (19.4)	23 (21.9)	37 (20.9)
History of STI			
No history of STI	78 (100)	141 (90.4)	219 (93.6)
History of STI	–	15 (9.6)	15 (6.4)
Screening for HIV			
screened for HIV	76 (33.5)	151 (66.55)	227 (97)
Not screened for HIV	2 (2.6)	5 (3.2)	7 (3)
Test result to HIV			
Reactive	2 (2.6)	5 (3.2)	7 (3)
Non-reactive	76 (97.4)	149 (96.8)	225 (97)

### Awareness and practice to IUCD

The most majority 76(97.4%) of cases and three-quarter (66%) of controls have heard about IUD. Seventy Nine point Seven Percent of cases and Thirty Four point Six Percent of controls has seen IUCD. Regarding partner discussion about modern contraception 79.2% of cases and 69% of controls have husband discussion.

**Fig. 1:**
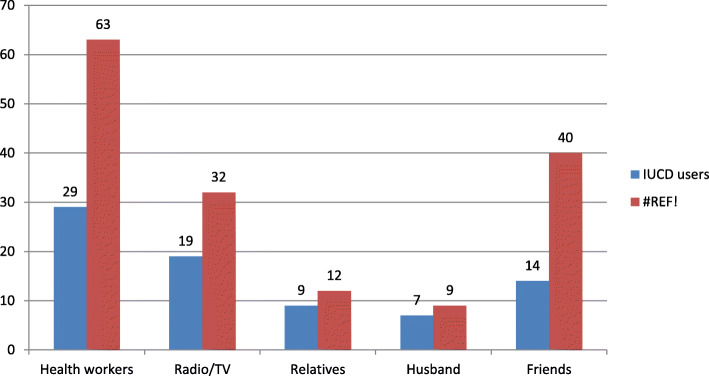
Source of information to modern contraception for women visiting family planning clinic at primary health care facilities in Mekelle city, Northern Ethiopia, Tigray region, 2017

The above graph shows that health workers are source of information for 29(37.2%) of cases and 63(40.4%) of controls followed by mass media for around quarter of (24.4%) of cases and friends for 40(25.6%) of controls (Fig. 1).

Among 234 respondents the majority of controls 110(70.5%) and less than one third use Injectable and OCP respectively.

### Health system factors

76.9% of cases and less than half (42.6%) of controls were told about the effectiveness of IUCD up to 12 years. Majority 85% of cases and around half (53.8%) of controls were told about the availability of health care providers able to insert IUCD.

### Factors associated with IUCD utilization

Variables considered for multiple logistic regressions were those with a *p*-value < 0.05 at bivariate logistic regression analysis and those variables included were age, occupation, marital status, ever heard about IUCD, Visual experience of IUCD, information given about (availability of IUCD, availability health care providers able to insert IUCD, protect pregnancy to 12 years and IUCD require minimal follow up after insertion.

Multiple logistic regression analyses identified that, marital status, number of pregnancy, number of alive children, Awareness about IUCD, visual exposure of IUCD and information given about the availability of Health Care provider able to insert IUCD were independent predictors that had statistically significant association with the current use of IUCD after adjusted for other covariates (Table 3).

**Table 3:** Determinants of IUCD utilization in Primary health care facilities in Mekelle city, Northern Ethiopia, Tigray region, 2017

Variables	Cases (78)(%)	Controls 156(%)	COR (95% CI)	AOR (95% CI)
Age (years)				
40–44	13 (16.7)	6 (3.8)	16.61 (5.05,54.65)*	7.23(.74, 70.79)
35–39	16 (20.5)	13 (8.3)	9.43 (3.44, 25.88)*	2.75(.46, 16.35)
30–34	17 (21.8)	26 (16.7)	5.01 (1.99,12.65)*	1.34(.26, 6.81)
25–29	23 (29.5)	42 (26.8)	4.19 (1.78, 9.93)*	1.14(.28, 4.65)
15–24	9 (11.5)	69 (44.3)	1	**–**
Occupation				
House wife	10 (12.8)	47 (30.1)	1.97 (0.57–6.78) *	0.1 (0.01–1.26)
Merchant	26 (33.3)	24 (15.4)	10.02 (3.11–32.33)*	0.62 (0.05–7.29)
Govt. Employee	23 (29.5)	29 (18.6)	7.34 (2.28–23.58)*	0.43 (0.04–4.89)
Private Employee	15 (19.2)	19 (12.2)	7.30 (2.13–25.08)*	0.65 (0.06–7.76)
Student	4 (5.1)	37 (23.7)	1	1
Marital status				
Married	73 (93.6)	101 (64.7)	7.95 (3.03–20.84)	8.59 (2.6–28.4) **
Single	5 (6.4)	55 (35.3)	1	1
Number of pregnancy (*N* = 194)				
≥5 times	14 (18.4)	10 (8.5)	4.94 (1.89–12.87)	5.7 (1.02–31.80) **
3–4 times	43 (56.6)	40 (34.2)	3.79 (1.95–7.39) *	2.04 (0.67–6.23)
1–2 times	19 (25)	67 (57.35)	1	1
Number of alive children (*N* = 177)				
> 2 children	42 (57.5)	42 (40)	5.50 (2.08–14.45)	3.5 (1.03–11.91) **
2 children	25 (34.2)	30 (28.6)	4.58 (1.65–12.70)	3.94 (1.14–13.55) **
1child	6 (8.2)	33 (31.4)	1	1
Ever heard about IUCD (234)				
Yes	76 (97.4)	103 (66)	19.55 (4.62–82.74)	14.15 (2.82–71.18)**
No	2 (2.6)	53 (22.6)	1	1
Have you ever seen IUCD (234)				
Yes	70 (89.7	54 (34.6)	16.53 (7.41–36.87)	10.4 (4.0–27.17) **
No	8 (10.3)	102 (65.4)	1	1
Information on availability of IUCD in PHCU				
Yes	73 (93.6)	120 (76.9)	4.38 (1.65–11.67)*	6.11 (0.55–68.02)
No	5 (6.4)	36 (23.1)	1	1
Information on availability of HCP that insert IUCD				
Yes	67 (85.9)	84 (53.8)	5.22 (2.56–10.63)	3.9 (1.5–9.7) **
No	11 (14.1)	72 (46.2)	1	1
Information on IUCD protect pregnancy for about 12 years				
Yes	60 (76.9)	66 (42.6)	4.49 (2.43–8.32) *	1.42(.33–6.15)
No	18 (23.1)	89 (57.4)	1	1
Information on IUCD requires less follow up				
Yes	35 (44.9)	105 (68.2)	2.63 (1.53–4.61) *	1.26(.36–4.39)
No	43 (55.1)	49 (31.8)	1	1

*Significant *p* < 0.25, and ** Significant *p* < 0.05

## Discussion

The percent distribution of IUCD among ever-married and currently married women is difficult to estimate since there is continuous shift from one method to the other. However, the type of selection of contraceptive method is related to the couple relationship status. By this study marital status is significant determinant of IUCD utilization, those women who are married are 8.6 times more likely to use IUCD than those single and divorced ([AOR (95%CI) =8.59(2.598–28.42)]. This finding is higher than studies conducted in Addis Ababa; Women who had married were 1.89 times more likely to use IUCD than women who are not married. It could be because of women who are married and in long term relation are more likely to choose IUCD for its ability to act as long term contraceptive methods, on the other hand those who are unmarried not think IUCD is necessary for their infrequent sexual activity or they may perceive their short term relation do not need the effect of IUCD. It also may be due to joined decision with their husband to prefer IUCD. But this finding is contrasted by study done based on Evidence from EDHS 2011, women who were temporarily live with their partners are about Two times more likely to use LARC([AOR (95%CI) =1.9(1.2,3.0) [15]. This may be a result of social and cultural in acceptability of giving birth before formal marriage at most part of Ethiopia.

Another strong predictor of IUCD utilization is awareness about IUCD 97% of users and 66% of non-users have ever heard about IUCD which implies those have awareness are about 14 times more likely to utilize the method than their counter parts ([AOR (95%CI) =14.2 (2.814–71.184). this is higher compared with the result of the 2 studies done in Mekelle town before 5 years that shows 55.3% of the respondents heard about IUCD and another evidence based study done Ethiopia which shows 48.5% of cases and 19.4% of controls have heard about IUCD [9, 15, 16]. The might be a result of continuous promotion and advertisement through media increases through time and the difference in the educational background of clients as the most of the clients are with secondary and tertiary education.

Number of alive children is the strong predictor for IUCD utilization, as this study implies those having > 2 children are more than three times more likely to use IUCD compared with women having a unit child [AOR (95%CI) =3.5 (1.03–11.9). This finding oppose with study done in India which states woman acceptance of IUCD are highest after first child (73.70%), 22.3% after two children and declined thereafter and 84% of users were in the peak child bearing age of 20–29 years this can be the resulted from women with increased parity may achieve desired family size and choose the most effective and permanent method (female sterilization) [17].

This finding is supported by studies done in Nepal and Hosanna towns. Comparative cross sectional study done in urban area of Nepal shows that women having more than 2 children are about 2 times more likely to utilize IUD compared with those having ≤2 children [AOR (95%CI) = 2.20 (1.12–4.32)] and other higher finding reported from case control study done on LACs utilization those who have 1–2 and 3–4 children are around six times to use IUCD compared with those hiving no child [AOR (95%CI) =5.5 (2.7, 11.0) and 6(3.0–12.0) [11, 15]. This positive association can be a result of logical governance of reproductive intentions by the total number of children and the discrepancy in Odds might be the difference in the sample size of the studies.

Information told about the availability of health care provider able to insert IUCD stay strong predictor in multivariate logistic regression. Those respondents who are told about this information are about four times more likely to use IUCD [AOR (95%CI) =3.89 (1.55, 9.74)].This finding is relatively lower compared with case control study conducted in Addis Ababa city, which states that those who provided with this information are five and half times more likely to utilize the method [AOR (95%CI) =5.765(1.646, 51.486) [18]. This can the problem with health care providers in counsel each aspects of IUCD and/or could be relatively higher awareness and sociodemographic difference between the Two Cities. It also might be a result of providers simply do not possess adequate knowledge about the IUD as a LARC method and consequently, provide incomplete information to potential clients or do not offer the IUD as a reliable method.

Visual exposure to IUCD is another independent predictor of IUCD utilization. Women who had ever seen IUCD are more than ten times to use IUCD than those with no visual exposure [AOR (95%CI) =10.4(4.0–27.17). This might be a result of health care providers neglect ion in counseling each option of contraception or lacking enough knowledge on counseling which is crucial step that helps clients to make a decision regarding voluntarily using a kind of most suitable, effective and safest contraceptive method. Or it can be due to a problem with promotion if IUCD through locally available audio-visual medias [15, 16].

One of the strength of this study was data was directly collected from respondents so there is no lost variable which determine IUCD use and no possible recall bias. As this is Case-control study compares those factors in each group (cases and control), so that cause-effect relation can be estimated. Although the readers should consider limitations like exclusion of private health care facilities, small sample size and lack of qualitative part which are recommended for further studies and innervations.

## Conclusion

The result of this study showed that being married, number of pregnancy, number of living children, awareness about IUCD, information given about the availability of health care provider able to insert IUCD and visual exposure of IUCD have positive association with IUCD utilization. Therefore stake holders like Tigray Regional health bureau, health care facilities and others should work for improvement in service quality and providers performance to increase the utilization of this effective and safe modern contraceptive method IUCD.

## Data Availability

The raw data documents are available upon request from the corresponding author.

## Abbreviations

CPR: Contraceptive prevalence rate
FMOH: Federal Ministry of Health
IUCD: Inra uterine contraceptive device
LARC: Long acting and reversible contraceptives
OCP: Oral contraceptive pills
PPHC: Public primary health care
TFR: Total fertility rate
